# When Somatic Delusions Meet Cyberchondria: An Unusual Case of Vitamin D Toxicity

**DOI:** 10.7759/cureus.85322

**Published:** 2025-06-04

**Authors:** Sally Namboodiri, Sanjay Rao

**Affiliations:** 1 Medicine, Louis Stokes Cleveland VA Medical Center, Cleveland, USA; 2 Radiology, Brigham and Women's Hospital, Harvard Medical School, Boston, USA

**Keywords:** acute hypercalcemia, celebrity doctor, cyberchondria, dietary supplement consumption, lack of trust healthcare, online health information seeking, renal failure, restrictive eating, somatic delusion, vitamin d toxicity

## Abstract

Patients with somatic delusional disorder are firmly convinced that something is wrong with their bodies and are often referred from one specialist to another without receiving a clear diagnosis. Some of these patients may turn to complementary or alternative medicine based on information found through online health searches. However, online health content is not regulated for quality, and much of it is inaccurate or potentially harmful, including fad diets and vitamin supplement advice from celebrity doctors. We present the case of a patient who believed he had Huntington’s disease and developed cyberchondria - compulsive online health searching - in an effort to treat his perceived condition. His distrust of conventional healthcare and reliance on unverified online sources led to a restrictive diet and excessive intake of dietary supplements, ultimately resulting in vitamin D toxicity, acute hypercalcemia, and renal failure. Managing patients with somatic delusions is challenging, as they often lack insight into their underlying psychiatric condition. A useful approach involves building a trusting relationship with a specialist familiar with the area of the somatic complaint. This relationship can help explore the patient’s symptoms and emotional stressors, eventually allowing for a gentle and gradual challenge to the delusional beliefs.

## Introduction

The internet enables individuals to access free health information without consulting a healthcare professional - a behavior known as online health information seeking (OHIS). An international survey conducted in 2018 found that 76% of respondents had engaged in OHIS within the previous three months [1]. A systematic review reported that OHIS can empower patients and potentially enhance the clinician-patient relationship [2]. However, OHIS can also become problematic, particularly in cases of cyberchondria, where individuals engage in compulsive health-related internet searches that increase anxiety and lead to adverse outcomes [3]. A further concern is the lack of regulation regarding the quality of online medical content. Patients may accept misinformation as credible, especially when it is accompanied by persuasive testimonials or endorsements from prominent physicians.

In this case report, we present a patient with a delusional belief that he had Huntington’s disease. He relied heavily on online sources, which convinced him that the condition could be cured through alternative therapies such as restrictive diets and excessive use of dietary supplements. These practices ultimately led to his hospitalization due to vitamin D toxicity.

## Case presentation

A 50-year-old male with a past medical history of oncocytoma status post left radical nephrectomy, dyslipidemia, benign prostatic hypertrophy, anxiety disorder, and delusional disorder - untreated by choice - and a family history of Huntington’s disease presented to the ED with abdominal pain and nausea that had been ongoing for three weeks. He reported that he had initially followed a paleo diet but switched to a ketogenic diet due to an “aversion to fats,” after which he developed non-radiating mid-epigastric abdominal pain. He believed the pain was triggered by food intake and noted that it resolved with self-initiated fasting. He denied emesis, diarrhea, constipation, fever, chills, blood in stools, melena, back pain, or hematuria. However, he reported decreased urinary output and an unintentional weight loss of 10 pounds over the same period.

His medications included tamsulosin and several over-the-counter dietary supplements, including vitamin D. He had numerous food allergies - including rice, coconuts, seeds, grains, apples, dairy, corn, lentils, squash, and beans - which he claimed caused symptoms such as “brain fog,” fatigue, tremors, or GI discomfort. He was unemployed and lived with his cousin. He denied tobacco, alcohol, or illicit drug use. His family history was notable for a mother and maternal grandmother who both died of Huntington’s disease in their 60s.

On presentation, he was afebrile with a pulse of 88, a blood pressure of 110/70 mmHg, a height of 68 inches, and a weight of 130 pounds - down from 142 pounds four months prior. Abdominal examination revealed mild tenderness in the mid-epigastric region without palpable masses. There was no costovertebral angle tenderness. Laboratory studies showed acute kidney injury, hypercalcemia, elevated vitamin D level, and suppressed parathyroid hormone level - findings consistent with vitamin D toxicity (Table 1).

**Table 1:** Laboratory results in the ED Four months prior to the ED visit, the patient’s creatinine was 1.2 mg/dL, calcium was 9.6 mg/dL, and vitamin D was 122 ng/mL.

Test	Result	Normal range
Blood urea nitrogen	38	8-20 mg/dl
Glucose	97	71-120 mg/dl
Creatinine	3.7	0.5-1.2 mg/dl
Estimated glomerular filtration rate	17	>/60 ml/min
CO₂	27	21-32 mmol/L
Calcium	12.5	8.5-10.1 mg/dl
Phosphorus	3.2	2.5-4.5 mg/dl
Vitamin D	258	30-75 ng/ml
Parathyroid hormone	<7	15-72 pg/ml

**Figure 1:**
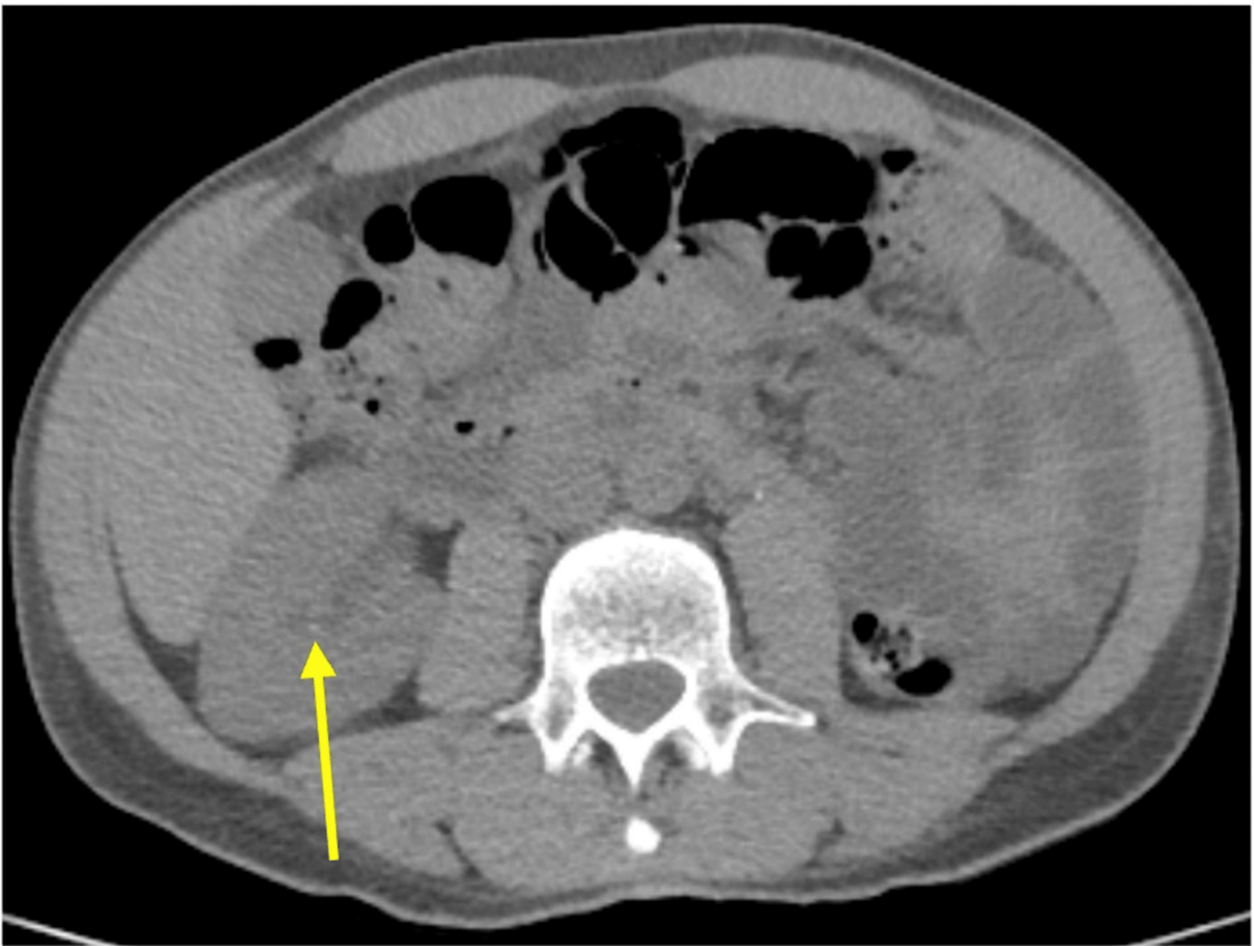
Non-contrast CT of the abdomen and pelvis showing a punctate calculus in the right interpolar calyx (yellow arrow) No associated hydronephrosis is observed in the solitary right kidney.

His non-contrast CT scan of the abdomen and pelvis revealed a minute non-obstructing calculus in the solitary right kidney, with no evidence of hydronephrosis (Figure 1).

The patient was admitted to the hospital. His post-void residual urine volume measured only 10 mL. The fractional excretion of sodium indicated a prerenal cause for his acute kidney injury. Vitamin D supplementation was discontinued, and he received 3 L of IV normal saline over 48 hours. His calcium and creatinine levels subsequently improved (Table 2).

**Table 2:** Laboratory results on days 1 and 2 of hospitalization and four weeks post-discharge

Test	Hospital day 1	Hospital day 2	Four weeks post-hospitalization	Reference range
Creatinine	3.7	2.6	1.8	0.5-1.2 mg/dl
Estimated glomerular filtration rate	17	26	40	>/60 ml/min
Calcium	12.5	9.4	9.3	8.5-10.1 mg/dl
Vitamin D	253	-	181	30-75 ng/ml

The patient claimed to have Huntington’s disease despite never undergoing genetic testing for the disorder. He described his hobby of researching online and watching TED Talks and YouTube videos about curing neurological diseases through dietary supplements, food restriction, and special diets. He cited a physician who reportedly cured her multiple sclerosis with diet changes and supplements. In this physician’s TEDx Talk, she explained that her doctors had no cure for her multiple sclerosis, so she turned to the internet and discovered research on the neurological benefits of the Paleo, or Hunter-Gatherer, diet. This diet provided vitamins and minerals at levels two to ten times the recommended daily allowance [4]. The patient also mentioned other online doctors who claimed that vitamin D was essential for brain health [5,6] and that certain foods could cause brain fog due to the “gut-brain” connection [7]. Based on this information, he avoided several foods and took 50,000 units of vitamin D daily for self-perceived “brain fog” symptoms for two months before admission. He then increased this dose to 100,000 units daily after experiencing worsening hand tremors one week before admission. He also consumed mega doses of other over-the-counter supplements, including calcium, folic acid, vitamin B12, copper, zinc, magnesium, potassium, and cassava root. During his hospital stay, he adamantly refused to see a mental health provider. At discharge, he was instructed to stop all dietary supplements, including vitamin D, and to follow up with his primary care practitioner (PCP).

Chart review revealed that the patient had been evaluated by a neurologist one month prior to admission, who found no evidence of Huntington’s disease based on normal neurological and cognitive exams. Genetic testing was offered, but declined by the patient. He also saw an endocrinologist for weight loss and told her that he distrusted traditional medical care, believing he could cure his Huntington’s disease through diet and supplements. He was unwilling to consider alternative explanations, such as a mental health disorder, for his symptoms. At his PCP visit one month after hospitalization, he reported switching to a high-creatine diet and starting hyperthermia treatments intended to increase heat shock protein levels to prevent neurological decline. His PCP warned that these practices could harm his kidney function. Although he was advised to stop all supplements and was counseled extensively on their potential harm, he continued taking them. His follow-up vitamin D level remained elevated one month after hospitalization (Table 2). The patient was subsequently lost to follow-up with his PCP and the healthcare system. Three years later, his PCP was notified of his death at age 53, but no information about the cause of death was available.

## Discussion

According to the Diagnostic and Statistical Manual of Mental Disorders, Fifth Edition (DSM-5), a person with delusional disorder exhibits one or more delusions for at least one month without significant functional impairment [8]. Somatic-type delusions, also known as monosymptomatic hypochondriacal psychosis, involve a fixed belief that something is seriously wrong with one’s body - whether it is an undiagnosed illness, an infestation, a dysmorphia, or body odor [9]. Many patients with somatic delusions repeatedly seek answers from multiple doctors and are often disappointed when no diagnosis is found; some even commit suicide due to despair over the lack of effective treatments. These patients generally lack insight and frequently refuse mental health care. Management of somatic-type delusional disorder has included antipsychotic and antidepressant medications, cognitive behavioral therapy, and electroconvulsive therapy, with varying degrees of success [10]. Our patient was convinced he had Huntington’s disease despite a neurologist informing him there was no evidence supporting this diagnosis. He self-reported mistrust of traditional medicine, and, although he admitted to anxiety about his health, he declined mental health care. Given that Huntington’s disease has no cure, he turned to the internet for answers. There, he encountered false information that reinforced his delusion and ultimately led to self-inflicted harm.

Cyberchondria is not formally recognized as a diagnosis in DSM-5 but can be considered a counterpart to hypochondriasis (illness anxiety disorder), which is recognized [2]. It is defined as anxiety related to online health searches and is characterized by compulsion, distress, excessive searching, reassurance-seeking, and mistrust of medical professionals when their opinions conflict with online information. Our patient met the criteria for cyberchondria. He admitted to compulsive OHIS, as demonstrated by his ability to quote numerous celebrity physicians’ websites and videos. He cycled between relief when finding a diet, supplement, or health practice that seemed to alleviate his perceived symptoms and anxiety when he felt a treatment was no longer effective. Research suggests an association between cyberchondria and the use of complementary and alternative medicine (CAM), indicating that patients with cyberchondria who do not receive adequate treatment from medical professionals may turn to CAM instead [11]; this was evident in our patient.

Vitamin supplements represent one form of CAM, with vitamin D being one of the most popular. The global vitamin D market was valued at 1.69 billion dollars in 2023 [12]. This market continues to grow despite the U.S. Preventive Services Task Force concluding there is insufficient evidence to assess the benefits and harms of screening for vitamin D deficiency in asymptomatic adults due to a lack of evidence supporting supplementation’s effect on fractures, falls, depression, diabetes, or cardiovascular disease [13]. The popularity of vitamin D is driven in part by online sources - websites, articles, and videos - promoting its benefits for memory issues, depression, fatigue, obesity, cardiovascular disease, and cancer; many involve celebrity physicians. Some websites recommend high doses of vitamin D as essential for immune health [14]. However, daily vitamin D supplementation exceeding 10,000 IU can lead to toxicity. Vitamin D toxicity is increasing, often due to errors in formulation, prescribing, or dispensing [15]. There are also cases of intentional use of very high doses by consumers unaware of the risks and believing that higher doses yield greater benefits [16]. Our patient took 50,000 to 100,000 IU of vitamin D daily to treat symptoms of his self-diagnosed Huntington’s disease, resulting in symptomatic vitamin D toxicity requiring hospitalization. Hypercalcemia developed due to increased bone resorption and enhanced intestinal calcium absorption, causing nausea and abdominal discomfort [17]. This hypercalcemia induced kidney injury via renal vasoconstriction and volume depletion secondary to nausea and increased urinary sodium excretion [18]. Chronic hypervitaminosis D led to nephrolithiasis due to elevated urinary calcium excretion (Figure 1). Vitamin D-induced hypercalcemia was promptly treated with IV fluids and cessation of vitamin D, which has a short half-life. Unfortunately, after hospitalization, our patient continued self-harming practices, including ongoing vitamin D use, a high creatine diet, and sauna treatments, all of which could worsen renal insufficiency. Although the cause of his premature death remains unknown, these practices may have contributed.

Patients with somatic delusional disorder who refuse mental health treatment may benefit from frequent visits with specialists, such as neurologists in our patient’s case, to build a therapeutic relationship [19]. If a neurologist had established an ongoing dialogue over several visits, the patient might have remained engaged in the healthcare system. A trusting clinician-patient relationship could enable gradual challenges to his delusion and the provision of safer alternatives to address his symptoms. Dismissing patients with somatic delusions as eccentric or “crazy” risks further alienation from medical care and poor outcomes. Our patient’s case illustrates that for individuals with somatic delusions and mistrust of healthcare, unregulated and false online health information can be dangerous, potentially leading to self-harm and death.

## Conclusions

Free health information is widely available on the internet but is not regulated for quality. Similarly, the billion-dollar vitamin industry allows patients to purchase supplements over the counter without clinician supervision. Online sources often promote the idea that high doses of supplements are more effective than standard doses while downplaying the risks of toxicity. Our patient, who had somatic delusional disorder, mistrusted the healthcare system because his self-diagnosed Huntington’s disease had no conventional cure. His reliance on false online information, largely from celebrity physicians, reinforced his belief that he could cure himself through risky dietary and supplement regimens. These choices ultimately resulted in acute kidney injury and vitamin D toxicity. Patients with somatic delusions and cyberchondria who refuse mental health care may benefit from regular visits with specialists relevant to their specific delusion. Such ongoing contact aims to build a therapeutic alliance, gradually challenge delusional beliefs, and offer safer alternatives to manage symptoms. In our patient’s case, establishing a relationship with a trusted clinician might have helped reduce his self-harming behaviors.
